# A New Algorithm for Identifying Cis-Regulatory Modules Based on Hidden Markov Model

**DOI:** 10.1155/2017/6274513

**Published:** 2017-04-11

**Authors:** Haitao Guo, Hongwei Huo

**Affiliations:** School of Computer Science and Technology, Xidian University, Xi'an, Shaanxi, China

## Abstract

The discovery of cis-regulatory modules (CRMs) is the key to understanding mechanisms of transcription regulation. Since CRMs have specific regulatory structures that are the basis for the regulation of gene expression, how to model the regulatory structure of CRMs has a considerable impact on the performance of CRM identification. The paper proposes a CRM discovery algorithm called ComSPS. ComSPS builds a regulatory structure model of CRMs based on HMM by exploring the rules of CRM transcriptional grammar that governs the internal motif site arrangement of CRMs. We test ComSPS on three benchmark datasets and compare it with five existing methods. Experimental results show that ComSPS performs better than them.

## 1. Introduction

Eukaryotic gene expression is regulated by cis-regulatory modules (CRMs). A CRM is a DNA sequence that contains multiple binding sites of one or more specific transcription factors (TFs) that regulate together an aspect of a gene expression pattern. A CRM typically has a length range from 100 to 2000 bp. Promoters, enhancers, and silencers are common CRM examples; they perform different regulatory functions and are located in 5′ flanking regions, 3′ flanking regions, promoter regions, intron regions, and even exon regions of regulated genes. The CRM discovery is the key to revealing mechanisms of gene regulation and understanding mechanisms of development, evolution, and disease.

Although CRMs play important roles in the regulation of eukaryotic gene expression, the discovery of CRMs [1, 2] remains a challenging problem in biology. The reasons are as follows. The distribution of CRMs in gene regulatory regions is very wide and some CRMs are even far from the target gene up to several hundreds of kbp; motif sites as functional elements of a CRM are short and degenerate, and their identification in itself is a difficult problem. Moreover, the complex regulatory structure of CRMs, which defines how motif sites within CRMs are organized to form regulatory complexes in what number, order, orientation, and spacing, further increases the difficulty of CRM discovery. CRMs of eukaryotic genes have the complex and varying structure; the motif sites within orthologous CRMs of different genes often mutate and rearrange, and the overall structures of CRMs are not conserved. Unfortunately, the internal regulatory mechanism of CRMs, which governs the organization of structure features, has not yet been fully understood. Thus, it is difficult to deterministically describe the regulatory structure of CRMs.

Current methods predict CRMs based on motif site clustering or modeling the regulatory structure of CRMs along the some clues. Although the overall CRM structure is not conserved, the biological reasons make the arrangement of motif sites within a CRM nonrandom, and the arrangement of these motif sites should be conducive to TFs binding. Many studies have shown that motifs are usually organized into composite elements (CEs) [3] to regulate eukaryotic gene expression. CEs are defined as functional units consisting of two or more motif sites that are located close to each other. The corresponding TFs interact with CEs to enhance or repress a specific transcriptional activation. The presence of a CE within a CRM constrains the ordering and spacing between motif sites within the CRM. Current CRM discovery methods can be divided into the following three categories according to CRM representation.

The first category simply views a CRM as a cluster of motif sites in windows and uses the set model to represent CRMs [4–9]. Some methods in this category employ statistical approaches to score clusters of motif sites in windows of a predefined given size and to identify the statistically significant site clusters as candidate CRMs. For example, MSCAN [4] uses *p* values to score clusters of motif sites in each window region. MotEvo [7] uses a Bayesian approach to calculate the posterior probabilities of all given motifs in each aligned window. Some other methods use combinatorial approaches to search a combination of motif sites satisfying specific constraints in a window region of a given size. For example, CMStalker [8] and CPModule [10] use a constraint satisfaction formulation and a constraint programming for itemset mining approach, respectively. The advantages of these methods are that they are simple, direct, and easy to implement. But they just define simple uniformity constraints for component motifs of CRMs and ignore the regulatory structure of CRMs, which is unrealistic. Moreover, their performance is usually limited by the window size setting and the hard specification of scoring thresholds.

The second category still regards CRMs as sets of motif sites but directly or indirectly constrains the order and distance of motif sites within CRMs based on phylogenetic conservation. Some methods search possible CRMs in orthologous sequences by looking for a group of predefined CRM patterns, that is, a set of specific motifs and constraints on ordering and intersite distances for these motifs; the corresponding methods are [11, 12]. Some other methods use sequences alignment, such as [13, 14], or motif site sequences alignment, such as [15], to detect conserved CRMs among species. Although this category of methods has achieved a certain degree of success, they are just applicable to the discovery of conserved CRMs in related species.

The third category uses probabilistic models to represent CRMs [16–23]. Most methods use Hidden Markov Models (HMMs) to model CRMs. The HMM can provide flexible structure representation by defining different states and transitions. Early methods, such as Cister [19] and Cluster-Buster [20], only define the distance constraint between motif sites but do not model any order between motif sites within a CRM. Subsequent methods, such as Stubb [21], BayCis [22] and CORECLUST [23], define transitions between motif sites to infer the possible spatial arrangement of motif sites within a CRM. The difference between these methods is that Stubb defines the correlation between some motifs, but, in inferring CRMs, it uses sliding window technology. The limitation of sliding window technology lies in the fact that the lengths of predicted CRMs are difficult to know in advance and are only based on experience to guess. BayCis and CORECLUST attempt to capture structural characteristics of CRMs by defining the correlation between all given motifs. This introduces a large number of model parameters to be estimated leading to a large number of computations.

This paper presents a new CRM discovery algorithm called ComSPS (the executable program of ComSPS is available at https://sites.google.com/site/onehoare/comsps). The algorithm still uses HMM to represent CRMs. Coexpressed genes often share common expression patterns and CRMs driving these expression patterns usually have an identical regulatory structure. We build a regulatory structure model based on HMM by exploring the rules of CRM transcriptional grammar [24] that governs the arrangement of motif sites within CRMs. These grammatical rules include not only motifs constituting CRMs but also the orientation preferences of motif sites, distributions of motif sites and their spacing, and the arrangement preferences of motif sites. Searching for the CRM transcriptional grammar is helpful to distinguish potential CRMs from background and thus improves the identification performance of CRMs. Moreover, modeling the CRM transcriptional grammars allows ComSPS to be able to analyze the CRM regulatory structure. In addition to motif states, ComSPS explicitly defines CRM states and can automatically determine sizes of CRMs. ComSPS uses the conservation of CRMs across sequences to improve the prediction accuracy without sequence alignment. We test ComSPS on three public benchmark datasets and compare it with five existing methods. Experimental results show that ComSPS performs better than them.

## 2. Materials and Methods

### 2.1. Overview

Binding sites of a TF usually mutate and are not identical in different sequences, but they have a common pattern known as a motif. We use a TFBS or a motif site to refer to an occurrence of a motif in sequences. Here, we use position weight matrices (PWMs) [25] to represent motifs.

The basic input of the algorithm includes a set of regulatory sequences, denoted by *X* = {*x*^1^, *x*^2^,…, *x*^*N*^}, which are from a group of coexpressed genes, and a set of motifs that are represented by PWMs, denoted by *W* = {*w*_1_, *w*_2_,…, *w*_*K*_}. These coexpressed genes are assumed to be regulated by similar CRMs with sites of motifs from* W*. In addition, the input includes some other model parameters. The algorithm outputs positions of found CRMs, along with TF labels of motif sites within the CRMs and their positions in sequences.

ComSPS employs an HMM to model the CRM regulation grammars shared by a group of coexpressed sequences. The specific regulation grammar rules introduced by the model include the number of motif sites, the origination of motif sites, the distance between motif sites, the order of motif sites, and the coassociation of motif sites. These rules are characterized by defining the parameters that are attached to the HMM model structure (state, state transition, and state transmission) and are determined by the learning of model parameters from given datasets. By the inference of the model, the CRMs following the predefined grammatical rules are deduced.

Specifically, states of motifs to probably be involved in the regulation and their reverse complementary counterparts are defined to capture motif sites constituting CRMs and their orientation preference; motif emission probabilities are described by PWMs to characterize the binding affinity of motifs; intermotif background states are defined to describe the distribution of nonmotif nucleotides and their duration distributions reflect the distance constraint between motifs; motif frequencies are added to the model to infer the distribution of motif sites; correlation probabilities are introduced to capture coassociated motif site pairs. The model is trained using an extended Baum-Welch algorithm based on the expectation maximization (EM) algorithm, and most parameters are adjusted automatically. The model uses the Viterbi algorithm to search CRMs by inferring the most likely state path. In addition, to improve the time efficiency of the algorithm, the training and inference of the model on all sequences are parallelized.

Before and after building the model, we performed some additional processing. If the given PWMs are directly from existing motif libraries (such as TRANSFAC [26] and JASPAR [27]) or outputs of third party de novo motif discovery methods, there may be more than one PWM describing motifs of a same TF, and these PWMs may have different lengths; however, the HMM architecture depends on the given PWMs, and many unnecessary states will be introduced when directly using them, thus increasing search space. In such cases, it is necessary to merge PWMs that represent the same TF before constructing the HMM. We further screened CRMs found by the HMM according to their conservation among all sequences to reduce false-positive predictions.

Based on these criteria, the whole algorithm can be simply described as follows:

- We filter the input PWMs based on clustering of similar PWMs. This step depends on the quality of the given PWMs, as optional preprocessing of the algorithm.
- For the input sequence, we use an HMM to model the regulatory structure of CRMs based on the filtered PWMs (or the given PWMs). We train model parameters using the Baum-Welch algorithm. Based on the trained model, we use the Viterbi algorithm to infer the locations of potential CRMs in sequences.
- For the found candidate CRMs, we further screen them based on their conservation among sequences and output them.

Each specific step of the algorithm is described in detail as follows.

### 2.2. PWM Filtering

To find the similar PWMs describing motifs of a same TF, we use a general clustering method. The basis of clustering is measuring the similarity between these PWMs. To reduce the impact of uncertainty and noise in biological data and to better measure the similarity between PWMs, we use the FISim algorithm [28] to calculate their similarity. FISim takes into account the relative importance of bases at different positions in a PWM and can give higher scores to more conserved positions, which makes FISim more robust than other methods. The following are specific steps of the filtering process:

- Use the FISim algorithm to measure the similarities between all given PWMs.
- Take similarities as weights, PWMs as vertices, and PWM pairs whose similarities are above a given threshold as edges to construct a weighted adjacency graph.
- Use the process [29] to find all dense subgraphs whose density are above a given threshold, and return them as the final clusters; let *K*′ be the number of clusters (*K*′ ≤ *K*). Each cluster corresponds to a unique TF.
- Choose the PWM whose length is closest to the average of all PWMs in a cluster as representative of the cluster. After filtering, the PWM set is denoted as *W*′ = {*w*_1_, *w*_2_,…, *w*_*K*′_}.

### 2.3. CRM Searching Based on the HMM

Based on the filtered PWM set *W*′ (if the given PWMs does not need filtering, then *W*′ = *W*), we build an HMM to identify CRMs in the given sequences. The HMM characterizes the regulatory structure of CRMs by introducing corresponding states and transitions.

#### 2.3.1. Model

The states of the constructed HMM, transition probabilities, and emission probabilities are defined as follows.

For a given sequence, it can be viewed as observations of the HMM. The HMM encodes the regulatory structure of the sequence according to a following hierarchical organization. A sequence can be considered as a mixture of CRMs which have variable lengths and are separated by inter-CRM background sequences; a CRM can be modeled as a concatenation of motif sites and intra-CRM background sequences.

**Figure 1:**
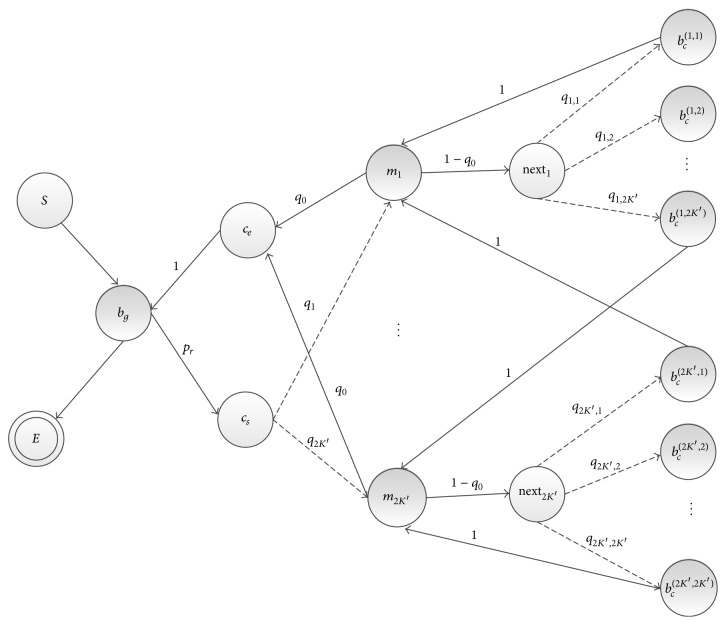
The ComSPS HMM state transition diagram. Emission states are represented by shaded circles. The auxiliary states are represented by circles, which are only used to model the structure (not to emit specific bases). *S* and *E* are the initiating and terminating states of the model, respectively. Only the transition probabilities marked by dotted lines need estimating in the model training.

As shown in Figure 1, inter-CRM backgrounds are denoted by the state *b*_*g*_; a CRM is denoted by the state *c*_*s*_, which indicates the start, and the state *c*_*e*_, which indicates the end. Here, *c*_*s*_ and *c*_*e*_ are auxiliary states. The auxiliary states do not emit specific bases and are only used to label the model structure; they are represented by circles in Figure 1. For the given sequence, the model assumes that it is regulated by at most *K*′ different TFs. The *K*′ states *m*_1_, *m*_2_,…, *m*_*K*′_ represent the corresponding motif sites, and PWMs of these motifs are from *W*′. Considering that motif sites may appear on the reverse complementary strand of the sequence, we define the reverse complementary of the motifs. We use *m*_*K*′+*i*_ to denote the reverse complementation of a motif *m*_*i*_. Thus, the model has 2*K*′ motif states. The model introduces an auxiliary state next_*i*_ to indicate that a motif state next to *m*_*i*_ is still within a CRM and to establish a transition of the motif *m*_*i*_ to other motifs to capture the correlation between them and motif frequencies. *b*_*c*_^(*i*,*j*)^ denotes spacers between sites of motifs *m*_*i*_ and *m*_*j*_, also known as intra-CRM backgrounds; when not specifically referring to spacers between two specific motifs, the superscript is removed and expressed as *b*_*c*_. The model assumes that emission probabilities of these background states follow the same distribution.

The transition probabilities are regarded as unknowns in the model and are defined as follows. For each position of the sequence, a decision is made to determine whether to initiate a CRM or generate a segment of background, from the CRM model with probability *p*_*r*_ or the background model with probability 1 − *p*_*r*_, respectively. If the model starts a CRM at the current position, then the current state becomes *c*_*s*_, indicating the start of a CRM. From the state *c*_*s*_, there is a probability *q*_*i*_ to initiate the CRM's first motif site *m*_*i*_, and each position in the following region with the length *l*_*i*_ has the same state *m*_*i*_, where *l*_*i*_ is the length of the motif *m*_*i*_. Then, the model transits to *c*_*e*_, with the probability *q*_0_, to end the CRM and return to the background *b*_*g*_; or the model can alternatively continue the CRM with the probability 1 − *q*_0_ and then chooses a next motif *m*_*j*_ with the probability *q*_*i*,*j*_, implanting the corresponding background *b*_*c*_^(*i*,*j*)^ between the sites of *m*_*i*_ and *m*_*j*_. Figure 1 shows the HMM architecture, with the transition probabilities marked at the arrows.

**Figure 2:**
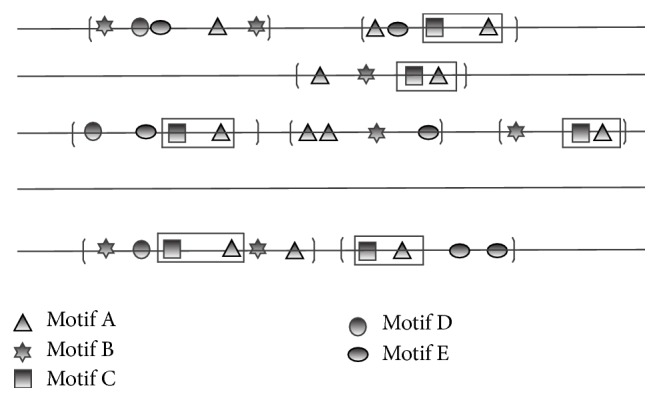
Correlation of coassociated motifs. In gene regulation, a TF usually works synergistically with other TFs by interactions to regulate a highly specific expression pattern. To facilitate such interactions, their binding sites are located adjacent to each other and form modules, also called composite elements (CEs) [3]. The same CEs perform similar functions in different genes, and they should be conserved in sequences and have a preferred arrangement. To capture coassociated motifs constituting CEs, the model defines the correlation probabilities. Based on CE' conservation assumptions, such motif pairs may cooccur repeatedly in regulatory sequences of genes, which are marked in rectangles in the example. The region within each pair of brackets represents a CRM. Each polygon represents a motif site within a CRM.

To capture the spatial correlations of coassociated motif sites as done in Stubb we introduce a parameter *r*_*i*,*j*_, which describes the probability that, along a DNA strand, a motif *m*_*j*_ site is located downstream of a motif *m*_*i*_ site and characterizes their specific arrangement, as shown in Figure 2. All motif state pairs are initialized to be noncorrelated; when the model detects that the cooccurrences of motifs *m*_*i*_ and *m*_*j*_ are statistically significant, the correlation between motifs *m*_*i*_ and *m*_*j*_ is identified and the parameter *r*_*i*,*j*_ is added to the model parameters. Under this definition, the transition probability between any motif sites is calculated as follows. If there exists correlation between *m*_*i*_ and *m*_*j*_, their transition probability *q*_*i*,*j*_ is *r*_*i*,*j*_; if they are not correlated, *q*_*i*,*j*_ is renormalized to ensure that ∑_*j*=1_^2*K*′^*q*_*i*,*j*_ = 1. Thus, the model only considers the correlation probabilities of the motif pairs which frequently cooccur and the estimated model parameters are reduced. Let *R*_*i*_ = {*j*∣ cooccurrence times of motifs *m*_*i*_ and *m*_*j*_ be statistically significant}, and *q*_*i*,*j*_ can be specifically expressed as

(1) $$\begin{array}{c} q_{i,j}=\left\{\begin{array}{cc} r_{i,j}, & j\in R_{i},\\ q_{j}\frac{1-\sum_{k\in R_{i}}q_{i,k}}{\sum_{k\notin R_{i}}q_{k}}, & j\notin R_{i}. \end{array}\right. \end{array}$$

To determine whether the cooccurrence of motifs *m*_*i*_ and *m*_*j*_ is statistically significant on a given sequence set, a *z*-score [21] for their cooccurrence times *T*_*ij*_ is defined as follows:

(2) $$\begin{array}{c} z_{ij}=\frac{T_{ij}-E_{ij}}{\sigma_{ij}}, \end{array}$$

where *E*_*ij*_ and *σ*_*ij*_ are the expectation and standard deviation of *T*_*ij*_, respectively. When *z*_*ij*_ and *E*_*ij*_ are greater than the given thresholds, the model determines the correlation between the two motifs.

The emission probabilities of the model are considered as known. In our HMM model, each state can emit a string of bases with variable length instead of a single base; this type of HMM is called the HMM with duration [30]. In the HMM, the probability of each state emitting a base sequence with a specific length is expressed as the product of the probability that the state generates any sequence of the length and the probability that the state generates the base sequence given the length.

Given a motif *m*_*k*_, its PWM and length *l*_*k*_ are known. Let *x*_1:*l*_*k*__ = *x*_1_*x*_2_ ⋯ *x*_*l*_*k*__ be a site of the motif and let *w*_*k*_[*i*, *j*] be the probability of base *i* at position *j* of its PWM. The probability generating the motif site is expressed as

(3) $$\begin{array}{c} P\left(\;x_{1:l_{k}}\;|\;m_{k},w_{k}\;\right)=\overset{l_{k}}{\underset{i=1}{\prod }}w_{k}\left[\;x_{i},j\;\right]. \end{array}$$

The inter-CRM background state *b*_*g*_ and the intra-CRM background *b*_*c*_ are modeled as the *k*th order and *k*′th order local Markov chains with the parameters *θ*_0_ and *θ*_1_, respectively. The two parameters are easily estimated from the given sequences. For the state *b*_*c*_, we assume that its length satisfies a geometric distribution with the expectation *h*, and the corresponding geometric distribution parameter *p*_*h*_ = 1/*h* is taken as a parameter of the algorithm to be specified in the configuration. Under the distribution, the probability of an intra-CRM background segment has a length *d* and is represented as follows:

(4) $$\begin{array}{c} P\left(\;d\;|\;b_{c},p_{h}\;\right)={\left(\;1-p_{h}\;\right)}^{d-1}p_{h}. \end{array}$$

For the state *b*_*g*_, its length reflects the distance between CRMs and is approximated by a geometric distribution with the expectation 1/*p*_*r*_.

#### 2.3.2. Inference and Training of the Model

Given a training set *D*, the model assumes that these sequences are independent. Therefore, the estimation of parameters can be done separately on each sequence. Let *λ* = {*p*_*r*_, *q*_0_, *q*_1_,…, *q*_*K*_,…, *r*_*i*,*j*_,…} and Θ = *W*′ ∪ {*θ*_0_, *θ*_1_}. In these parameters, other parameters except for *r*_*i*,*j*_ are derived from the existing HMM. To get *r*_*i*,*j*_ from a *Q* function, we follow the same process as done in [31]. Specifically, *r*_*i*,*j*_ is estimated on the training set as follows:

(5) $$\begin{array}{c} r_{i,j}=\left\{\begin{array}{cc} \frac{T_{ij}}{\sum_{k\in R_{i}}T_{ik}}, & R_{i}^{\prime }=⌀,\\ \frac{T_{ij}}{\sum_{k\in R_{i}^{\prime }}T_{ik}}\left(1-\underset{k\in R_{i}^{\prime }}{\sum }r_{i,k}\right), & R_{i}^{\prime }\neq ⌀, \end{array}\right. \end{array}$$

where *R*_*i*_′ is the complement of *R*_*i*_ and *T*_*ij*_ are cooccurrence times of motifs *m*_*i*_ and *m*_*j*_ on the given training set. Under the assumption of sequence independence, *T*_*ij*_ is the sum of cooccurrence times *T*_*ij*_(*x*) of motifs *m*_*i*_ and *m*_*j*_ on each sequence *x*: *x* ∈ *D*.

*E*_*ij*_ and the second term *E*(*T*_*ij*_^2^) of *σ*_*ij*_ can be estimated on the training set *D* as follows:

(6) Eij∑x∈DTijxPx ∣ λ=∑x∈D ∑πTijx,πPx,π ∣ λ,ETij2E∑x∈D ∑kIijkx2=E∑x∈D ∑kIijk2x+2∑x∈D ∑k,k′>kEIijkx,Iijk′x=E∑x∈D ∑kIijkx+2∑x∈D ∑k,k′>kPIijkx,Iijk′x,

where *π* is a state path and *I*_*ijk*_(*x*) is the indicator variable for the event where motif *m*_*i*_ is followed by motif *m*_*j*_ located at position *k* in a sequence *x* of the training set *D*.

To estimate *λ*, the Baum-Welch algorithm is extended to maximize the likelihood log⁡*P*(*D*∣*λ*, Θ). For a sequence *x* ∈ *D*, we define *π* and *d* as a state path of the model and the state duration sequence, respectively. Finally, log⁡*P*(*D*∣*λ*, Θ) is represented as follows:

(7) log⁡PD ∣ λ,Θ∑x∈Dlog⁡Px ∣ λ,Θ=∑x∈D ∑π,dlog⁡Px,π,d ∣ λ,Θ.

To maximize the likelihood log⁡*P*(*D*∣*λ*, Θ), we turned to maximize the *Q* function of log⁡*P*(*x*, *π*, *d*∣*λ*, Θ) following the process in [31]. The *Q* function is solved by the EM algorithm to iteratively update *λ* and finally converge to a locally optimum *λ*.

Based on the trained model, we use the Viterbi algorithm [31] to infer the most possible state path in each given sequence to be searched. The algorithm finds a group of CRMs in the sequences. For a given CRM *c*, we give a score to it by a log likelihood ratio as follows:

(8) $$\begin{array}{c} LLR\left(\;c\;\right)=\log\frac{P\left(c\;|\;\theta_{1},\lambda ,W^{\prime }\right)}{P\left(c\;|\;\theta_{0}\right)}, \end{array}$$

where *P*(*c*∣*θ*_1_, *λ*, *W*′) and *P*(*c*∣*θ*_0_) are the probabilities of *c* generated by the CRM and background models, respectively.

#### 2.3.3. Implementation

The implementation is as follows:

- In the determination of the correlation between motifs, thresholds of the *z*_*ij*_ score and the expectation *E*_*ij*_ are set to 1.
- In the model, for two local Markov chains used by background models, the default values of their orders *k* and *k*′ are 1 and 2, respectively, and their parameters *θ*_0_ and *θ*_1_ are off-line calculated in a bookkeeping way.
- Considering the fact that data sparsity can lead to overfitting, for the probability *p*_*r*_ to initiate a CRM and the probability *q*_0_ to terminate a CRM, they are specified in the configuration without using the Baum-Welch algorithm for estimation. In the model, *p*_*r*_ and *q*_0_ are set to 0.001 and 0.1, respectively, which works well in most cases. For the expectation *h* of the distance between motif sites within a CRM, *h* is set to 50 as a default; for a CRM with dense clustering of motif sites, *h* is set to a small value, such as 20.
- To improve time performance of ComSPS, we perform parallel optimization for the training and inference processes of the model. Since the model assumes that all sequences are independent, some steps of the two processes on a set of sequences can be performed concurrently. Specifically, for the model training, during each iteration, each of the calculations for expectations of state transition counts and the revaluation of sequence likelihoods can be executed concurrently on each sequence. The inferring process on each sequence is an independent task and thus it can be simultaneously performed on multiple sequences. In the concrete implementation, we perform the parallel acceleration by multithreading technology.

### 2.4. CRM Screening

Before further processing, the CRMs predicted by the HMM are first filtered according to a weight threshold *w*_*c*_, leaving CRMs above the threshold as candidate CRMs. The threshold *w*_*c*_ is set in the configuration file. These CRMs fit to the model and are statistically significant. Real CRMs should be conserved across multiple genes. So we further screen the candidates based on their conservation among given sequences. This step is executed only when the sequence set to be searched contains more than three sequences with predicted CRMs.

**Figure 3:**
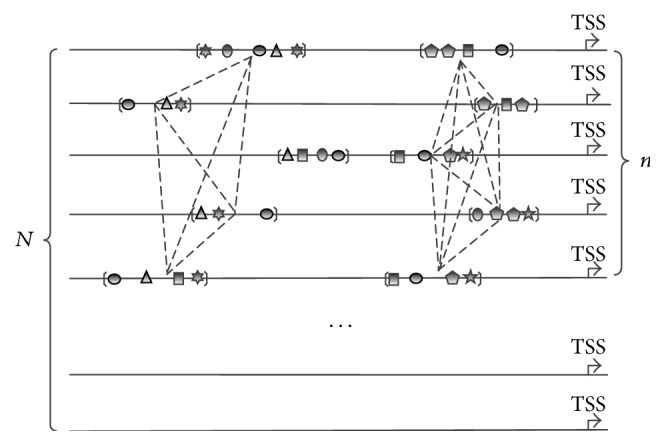
An example of clusters of CRMs. Given *N* coexpressed sequences, it is assumed that there are two types of CRMs to be predicted in *n* sequences. CRMs belonging to a same type constitute a clique. The region within each pair of brackets represents a CRM. Each polygon represents a motif site within a CRM. Dotted lines connect sufficiently similar CRMs (above a given threshold). The arrow indicates the gene start site.

To explore the conserved CRMs between multiple sequences, we integrate all information on similarities between CRMs into one graph. In the graph, each node represents a CRM, and an edge between two nodes indicates that the corresponding CRMs coming from different sequences are sufficiently similar (their similarity score is above a given threshold). According to the definition, the node sets containing CRMs conserved across multiple sequences form a clique in the graph, and a maximum clique of the graph corresponds to a possible type of CRMs, as shown in Figure 3. Therefore, the steps of identifying conserved CRM can be described briefly as follows.

Firstly, an undirected adjacency graph to describe the similarity relation between CRMs is constructed. In the model, the similarity between two CRMs is scored based on the contained motifs and motif sites without considering intra-CRM background. Specifically, the similarity score *D*(*c*_*i*_, *c*_*j*_) between two CRMs *c*_*i*_ and *c*_*j*_ is defined as follows:

(9) $$\begin{array}{c} D\left(\;c_{i},c_{j}\;\right)=\mu_{m}\frac{\left|M_{c_{i}}\cap M_{c_{j}}\right|}{\left|M_{c_{i}}\cup M_{c_{j}}\right|}+\left(\;1-\mu_{m}\;\right)\frac{\left|{MS}_{c_{i}}\cap {MS}_{c_{j}}\right|}{\left|{MS}_{c_{i}}\cup {MS}_{c_{j}}\right|}, \end{array}$$

where *μ*_*m*_ is a weight coefficient as a configurable parameter, *M*_*c*_*i*__ and MS_*c*_*i*__ are the motif set and the motif site set of a CRM *c*_*i*_, respectively.

Then, the process [29] is performed to enumerate all the maximal cliques with three or more than three nodes.

Lastly, we combine these found CRM cliques and then output all CRMs in these cliques. The conservation CS(*c*_type_) of a type of CRMs *c*_type_ from a clique with the node set *U* can be scored as follows:

(10) $$\begin{array}{c} CS\left(\;c_{type}\;\right)=\frac{1}{N}\underset{i,j\in U;i<j}{\sum }D\left(\;c_{i},c_{j}\;\right), \end{array}$$

where *N* is the sequence number of the whole sequence set.

## 3. Results and Discussion

We selected five compared methods, CPModule [10], MotEvo [7], Cluster-Buster [20], Stubb [21], and BayCis [22], and evaluated these methods on three public benchmark datasets: XIE dataset [32], Muscle dataset [33], and REDfly dataset [34]. Using public benchmark datasets is more convenient to measure the similarity between the prediction results of the methods and the actual results and objectively evaluate their performances.

### 3.1. Performance Evaluation

Given sequences to be searched, all CRM discovery methods output positions of predicted CRMs. The prediction of a method for CRMs can be viewed as classifying for the base at each position in the sequences. The bases are predicted to belong to a CRM, and they are annotated as Positive (P); the bases are predicted to belong to background, and they are annotated as Negative (N). Each annotation is either True (T) or False (F); thus, corresponding annotations are divided into four categories. Counting each category of annotations, they are denoted as nTP, nFP, nTN, and nFN. Obviously, the more true annotations of nTP and nTN relative to the false annotations of nFP and nFN, the higher the similarity between the prediction result and the actual result.

To quantify the similarity, some measures are designed. Let nPP (Predicted Positives) represent the number of bases that are predicted as CRMs, where nPP = nTP + nFP. Let nAP (Actual Positives) represent the number of bases that the actual CRMs contain, where nAP = nTP + nFN. The ratios of nTP to nPP and nAP define two important measures, sensitivity (Sn) and precision (Pr), respectively.

However, from their definitions, we can easily see that the two measures oppose each other. In this sense, if a method is expected to highlight a single measure by only changing the tightness of the search conditions but keeping the search strategy, it will inevitably decrease the other measure. Thus, to comprehensively evaluate the performance of a method, it needs to consider them together, for example, using P/R curve analysis or introducing balanced measures of Sn and Pr, such as F1-score and ASP.

In particular, the correlation coefficient (CC) as a common overall performance measure is often used to measure statistical correlation between the prediction results and the actual results. As a special case of the Pearson correlation coefficient of two variables, it was introduced by Burset and Guigó [35] to evaluate gene structure prediction and later widely used in other aspects of bioinformatics. The value of CC ranges between −1 and +1.

(11) CC=nTP×nTN−nFN×nFPnTP+nFNnTN+nFPnTP+nFPnTN+nFN.

All the above measures are based on the classification for bases, and they are referred to as the measures at base (or nucleotide) level. A similar classification can be made at motif site level, when an evaluated method gives all motif site information within predicted CRMs. However, since not all methods give the specific motif sites within CRMs, our evaluation is only limited at base level.

For the experiment on the XIE and Muscle datasets, we used CC as the measure to evaluate the methods. For the experiment on the REDfly dataset, we evaluated the methods according to the evaluation protocol of Ivan et al. [34]. The evaluation protocol is simply described as follows. On each subdataset, the average length of CRMs in the subdataset is calculated and then provided to CRM discovery methods as a parameter. These methods are required to output the CRMs closest to the length. Limited by this evaluation framework, it has nPP = nAP; thus, Sn = Pr. Hence, it can use only Sn as the measure. Furthermore, to evaluate the statistical significance of the prediction results, an empirical *p* value is introduced. Specifically, the empirical *p* value is defined as the probability that Sn of a stochastic prediction is greater than that of the prediction result. This *p* value is calculated by using the stochastic simulation. When the *p* value of Sn is less than 0.05, the prediction is considered statistically significant.

### 3.2. XIE Benchmark

#### 3.2.1. XIE Dataset

The dataset was constructed by Xie et al. [32]. The dataset contains 22 sequences. Each of these sequences is 1000 bp in length and they are from human, chicken, and mouse genomes. Among these sequences, there are 20 sequences with each containing an implanted CRM and two sequences containing no implanted CMRs. These CRMs have the length of at most 164 bp and contain binding sites of three TFs: Oct4, Sox2, and FoxD3. The distance between these TFBSs follows a Poisson distribution with expectation of 10.

The benchmark constructed four PWM test sets by adding different number of decoy PWMs. Specifically, noise ratios of these PWM test sets are 7/10, 17/20, 27/30, and 37/40, respectively. Each PWM test set contains 10 collections independently sampled from 516 TRANSFAC PWMs. This dataset can be downloaded from the web site provided by [9].

#### 3.2.2. Testing Results

On this dataset, following the way of three other HMM-based methods, Cluster-Buster, Stubb, and BayCis, all sequences are used as the training set and the testing set simultaneously for ComSPS. For Stubb, CPModule, and MotEvo, which depend on window size settings, they were tested with three window size settings, 100 bp, 150 bp, and 200 bp, which are around the average length of CRMs. Other parameters of all these methods remain default.

**Figure 4:**
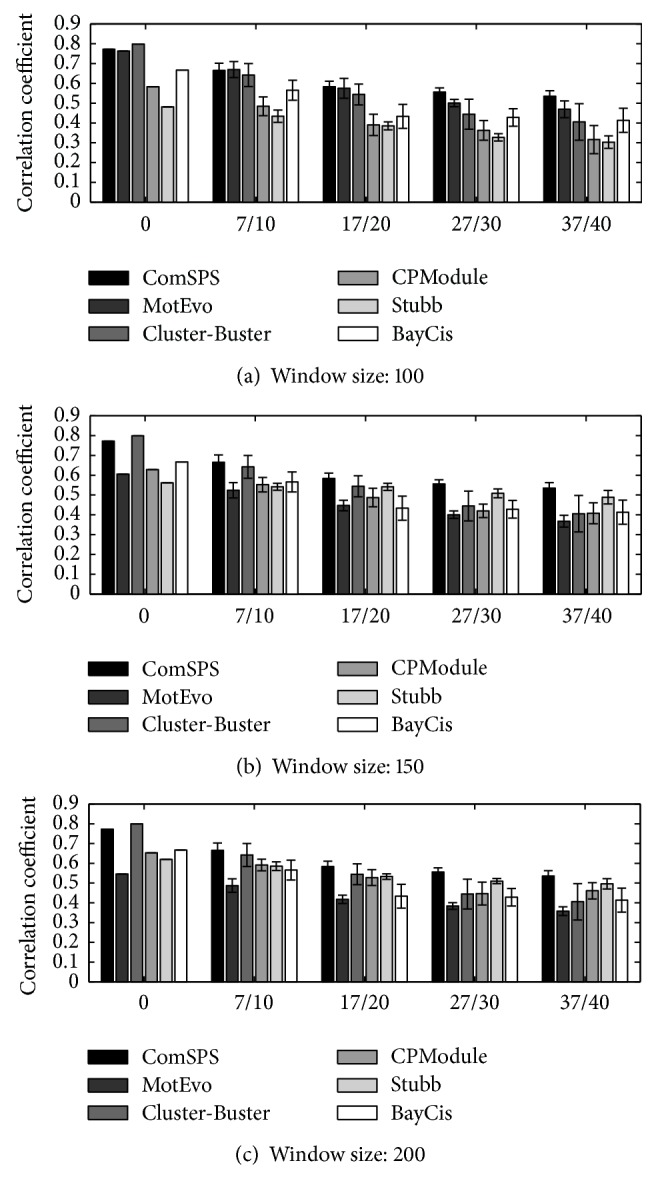
CC performances of all methods at different noise levels on the XIE dataset.

Figure 4 shows the means and variances of CCs of all methods at different noise levels on the dataset. Overall, ComSPS performed more stably than other methods and made the best predictions at different noise levels.

The figure also shows that, with increasing noise, the performances of all methods inevitably decreased, and their sensitivities to noise revealed great differences. ComSPS and Stubb performed the most stably, while Cluster-Buster was most sensitive to noise. Moreover, at the same noise level, these methods also showed a consistent trend for the collections of different decoy PWMs. The window clustering methods showed different performance trend for different window size settings. MotEvo seemed to perform better for small window size settings and CPModule was the opposite, while Stubb tended to perform best with the window size setting of 200 bp close to the average length of CRMs on the dataset. This may be the result of their different scoring or search strategies for CRMs.

### 3.3. Muscle Benchmark

#### 3.3.1. Muscle Dataset

The dataset was initially compiled by Wasserman and Fickett [36]. Later, it was extended by Klepper et al. [33] and used for the evaluation for CRM discovery methods in [33]. The dataset contains 24 sequences, as well as five motifs (Mef2, Tef, Srf, Sp1, and Myf), which play an important role in muscle regulation. The 24 genomic sequences are from mouse, cow, rat, chicken, and human, and their lengths range from 269 bp to 1000 bp (the average is 851 bp). The dataset has one CRM in each sequence. These CRMs range in length from 14 bp to 194 bp, with an average of 120 bp. Each CRM contains two to eight motif sites (the average is 3.5). The dataset and CRM annotations are taken from the companion web site of [33].

#### 3.3.2. Testing Results

For ComSPS, all sequences are used as the training and testing sets simultaneously consistent with other three HMM-based methods, Cluster-Buster, Stubb, and BayCis. For the window clustering methods, we chose four different window sizes, 100 bp, 150 bp, 200 bp, and 300 bp, which are close to the median, mean, third quartile, and the maximum value of CRM lengths on the dataset, respectively. Other parameters of all these methods remain default.

**Figure 5:**
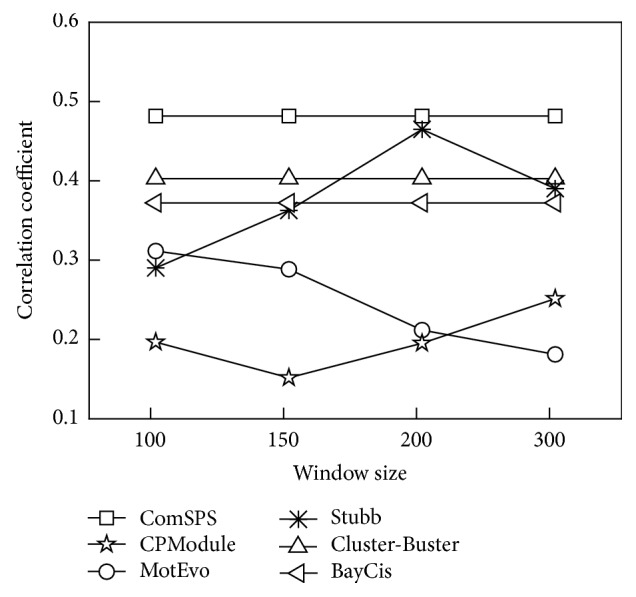
CC performances of all methods at different window sizes on the Muscle dataset.

Counting all TPs, TNs, FPs, and FNs predicted by each method on each sequence, we calculated their CC scores on the whole sequence set. Moreover, for the window clustering methods, we calculated the CC scores under each window size setting. The results are shown in Figure 5.

On this dataset, the prediction performance of ComSPS significantly outperformed the other methods. Overall, the HMM-based methods were superior to window clustering methods. Specifically, Cluster-Buster and BayCis made good predictions, which are second only to ComSPS. The window clustering methods showed a similar trend to that on the XIE dataset and still had a greatly different performance under different window settings. For example, Stubb made a good prediction close to ComSPS under the 200 bp window setting but performed slightly better than the lowest CPModule under the 100 bp window setting.

#### 3.3.3. Cross Validation

To evaluate the ability of ComSPS to learn the parameters, we performed 10-fold cross validation on the dataset. Since other HMM-based methods cannot explicitly specify the training set and the test set, we only verified ComSPS. The CRM prediction is not a simple binary classification task. The structural flexibility of CRMs leads to the great difference in performance of methods even to identify different occurrences of similar CRMs in different sequences, as shown in Table 1. As a result, variation in the prediction performances of methods when using different training sets will be obscured by inherent differences in prediction performance for different occurrences of CRMs. We performed 10 times 10-fold cross validation by shuffling and splitting the given sequence set into different training and testing sets.

As shown in Table 1, the variance of the performances of ComSPS on most of sequences was small, which indicates that ComSPS was not sensitive to varying training sets. So it demonstrated that the process of parameter estimation of ComSPS stably converges to some values around a local optimum in most cases.

### 3.4. REDfly Benchmark

#### 3.4.1. REDfly Dataset

Compared to the XIE and Muscle datasets, the REDfly dataset [34] is a larger dataset and it contains longer sequences and more complex CRMs. The dataset contains a total of 33 subdatasets. These subdatasets contain 53 PWMs and 719 sequences in total. Each subdataset has 4–77 sequences, with an average of 16, and these sequences have a total length of about 5.7 Mbp. On each subdataset, each sequence contains only one CRM. Each CRM has a different length and overall their average lengths have a range of 442–1248 bp. These CRMs perform functions in the early development of* Drosophila* and their annotations are derived from the REDfly database [37]. For the statistics on each subdataset, please refer to Table  1 in [34].

#### 3.4.2. Testing Results

On this dataset, for ComSPS, all sequences were used simultaneously as the training set and the test set. For the methods that depend on a window size, according to evaluation protocol in [34], we precomputed the average length of real CRMs on each subdataset and provided the length for the methods as a predetermined window size parameter. These methods outputted CRMs that have the length of the specified window size and have the highest scores as the final prediction results. Other methods still maintained their default settings.

Sensitivities and empirical *p* values of the prediction results of all methods are shown in Table 2. The results indicate that ComSPS performed very well on 16 of the 33 subdatasets, was superior to other methods, and made significant predictions on approximately half of subdatasets.

In its all statistically significant predictions (*p* value ≤ 0.05), the range of the sensitivity of ComSPS is 15–56%, with an average of 26%. Since the evaluation protocol limits the length of CRMs to be predicted, 100% sensitivity was very hard to get. Actually, from the table, we can see that the maximum possible average sensitivity on all subdatasets is approximately 77%. It assumes that there is a 100 bp CRM to be predicted in a 1000 bp sequence. The average sensitivity of 26% means that the overlap of a predicted CRM with the real CRM is about 26 bp. According to the definition of the maximum possible sensitivity, the real CRM has 77 bp to be predictable. Thus, in the sense, the overlap of the prediction of ComSPS with it is more than 1/3. From a biological point of view, this resolution is sufficient to recover real CRMs. Specifically, we can first roughly locate CRMs by ComSPS and then accurately determine the specific positions of CRMs by experimental means.

Regarding other methods, their prediction performances were greatly different on the dataset. Stubb and MotEvo showed excellent prediction performance, as they made statistically significant predictions on 12 and 10 subdatasets, respectively; BayCis and CPModule made statistically significant predictions on 8 and 7 subdatasets, respectively; Cluster-Buster performed worst and made random predictions on most of the subdatasets.

## 4. Conclusions

CRMs play an important role in the transcriptional regulation of eukaryotic genes, and their identification is the key to understanding the mechanisms of gene transcription regulation. To improve the identification performance of CRMs, this paper presents a new CRM discovery algorithm from the perspective of exploring the rules of CRM transcriptional grammar to build a regulatory structure model of CRMs. Experimental results revealed that the proposed algorithm performed better than compared methods on these tested benchmark datasets.

CRM discovery algorithms have been developed for many years and have experienced great progress, but they are far from being mature and still require further improvement. Currently, chromatin immunoprecipitation sequencing (ChIP-Seq) technology provides a large amount of data that can be used for the identification of motifs and CRMs. With the help of these new data, we believe that the prediction accuracy for CRMs can be further improved. However, these data are often short in length and huge in number; thus, they bring new challenges to existing methods. After processing ChIP-Seq results, the data analysis requires special algorithms. Developing new algorithms that are able to effectively identify CRMs from ChIP-Seq data is the focus of our future research.

## Figures and Tables

**Table 1:** The mean and standard deviation (STD) of CC performance on single gene on the muscle dataset.

Sequence name	CC mean (STD)
M13631	0.92 (0.00)
J04971	0.37 (0.04)
K01464	0.93 (0.00)
M20543	0.46 (0.15)
M21390	0.82 (0.02)
M22381	0.00 (0.00)
J04699	0.15 (0.01)
M57905	0.43 (0.00)
X14726	0.89 (0.09)
X59034	0.00 (0.00)
V01218	0.70 (0.01)
M63391	0.38 (0.04)
X12971	0.35 (0.16)
M95800	0.42 (0.03)
L21905	0.00 (0.00)
X05632	0.15 (0.29)
U02285	0.38 (0.13)
M62404	0.62 (0.05)
X62155	0.36 (0.01)
M13483	0.53 (0.00)
M84685	0.00 (0.00)
X73887	0.42 (0.04)
X67686	0.89 (0.00)
U18131	0.00 (0.00)

**Table 2:** The performances of all methods on the REDfly dataset.

Data Set	#Seq, Length, Max. Sens.^*∗*^	ComSPS^†^	MotEvo^†^	Cluster- Buster^†^	CPModule^†^	Stubb^†^	BayCis^†^
mapping1.adult mesoderm	34/254800/0.71	0.34 (0.14)	0.63 (0.05)	0.32 (0.15)	1.00 (0.00)	0.51 (0.05)	0.63 (0.05)
mapping1.amnioserosa	5/28085/0.76	**0.00 **(0.43)	0.13 (0.13)	0.31 (0.09)	0.09 (0.16)	0.25 (0.15)	1.00 (0.00)
mapping1.blastoderm	7/49635/0.84	**0.02 **(0.15)	**0.00 **(0.32)	0.76 (0.07)	**0.02 **(0.15)	**0.00 **(0.36)	**0.04 **(0.14)
mapping1.cardiac mesoderm	77/698840/0.77	**0.00 **(0.26)	0.09 (0.15)	0.42 (0.07)	0.65 (0.02)	0.08 (0.22)	0.60 (0.03)
mapping1.cns	8/42979/0.76	0.94 (0.04)	0.51 (0.10)	0.15 (0.14)	**0.04 **(0.17)	0.48 (0.10)	0.63 (0.08)
mapping1.dorsal ectoderm	34/352108/0.80	0.55 (0.08)	0.17 (0.17)	**0.04 **(0.24)	0.58 (0.08)	0.08 (0.22)	0.90 (0.00)
mapping1.ectoderm	8/67490/0.77	0.72 (0.07)	**0.02 **(0.16)	1.00 (0.02)	0.09 (0.13)	**0.01 **(0.20)	**0.01 **(0.21)
mapping1.endoderm	37/311000/0.72	**0.00 **(0.47)	0.09 (0.15)	0.44 (0.08)	0.82 (0.03)	**0.01 **(0.24)	0.67 (0.05)
mapping1.eye	51/416473/0.74	**0.01 **(0.31)	0.46 (0.07)	1.00 (0.00)	**0.01 **(0.28)	1.00 (0.00)	0.38 (0.12)
mapping1.fat body	16/92723/0.82	0.14 (0.21)	1.00 (0.00)	0.57 (0.04)	0.37 (0.12)	0.14 (0.20)	0.37 (0.12)
mapping1.female gonad	6/49494/0.70	0.59 (0.04)	**0.00 **(0.33)	1.00 (0.00)	0.49 (0.05)	**0.03 **(0.24)	**0.02 **(0.26)
mapping1.glia	18/156531/0.69	**0.00 **(0.56)	0.19 (0.13)	1.00 (0.00)	0.09 (0.18)	0.49 (0.09)	1.00 (0.00)
mapping1.imaginal disc	5/22831/0.93	**0.00 **(0.19)	0.53 (0.07)	0.45 (0.08)	0.18 (0.10)	0.55 (0.09)	**0.00 **(0.19)
mapping1.male gonad	10/44269/0.62	**0.03 **(0.24)	0.12 (0.17)	0.48 (0.07)	1.00 (0.00)	0.22 (0.15)	0.55 (0.05)
mapping1.malpighian tubules	7/63008/0.82	0.24 (0.12)	**0.02 **(0.28)	1.00 (0.00)	0.40 (0.04)	0.10 (0.25)	1.00 (0.00)
mapping1.mesectoderm	47/441597/0.77	0.58 (0.05)	0.14 (0.21)	0.63 (0.03)	**0.05 **(0.30)	0.18 (0.20)	0.63 (0.03)
mapping1.mesoderm	12/149915/0.80	**0.05 **(0.19)	**0.00 **(0.35)	0.69 (0.06)	0.47 (0.10)	**0.02 **(0.21)	0.34 (0.12)
mapping1.neuroectoderm	69/616635/0.76	**0.02 **(0.21)	**0.00 **(0.36)	0.39 (0.06)	**0.01 **(0.23)	**0.01 **(0.34)	**0.05 **(0.16)
mapping1.pns	8/69044/0.85	0.37 (0.11)	**0.01 **(0.22)	0.50 (0.09)	0.35 (0.11)	**0.03 **(0.19)	0.39 (0.10)
mapping1.salivary gland	4/31338/0.81	0.27 (0.08)	0.13 (0.12)	0.60 (0.01)	0.61 (0.01)	0.55 (0.06)	0.21 (0.10)
mapping1.somatic muscle	5/45712/0.83	0.29 (0.14)	0.60 (0.08)	0.41 (0.11)	0.55 (0.09)	0.29 (0.12)	0.13 (0.18)
mapping1.tracheal system	16/87140/0.72	0.71 (0.05)	0.55 (0.08)	0.70 (0.05)	0.74 (0.04)	0.55 (0.08)	1.00 (0.00)
mapping1.ventral ectoderm	45/233441/0.75	**0.01 **(0.23)	**0.00 **(0.31)	0.17 (0.13)	0.11 (0.15)	**0.00 **(0.38)	0.44 (0.08)
mapping1.visceral mesoderm	7/40315/0.80	**0.04 **(0.19)	0.06 (0.18)	1.00 (0.00)	0.64 (0.05)	0.46 (0.10)	0.64 (0.05)
mapping2.ectoderm	54/534081/0.78	**0.02 **(0.16)	0.08 (0.14)	0.08 (0.14)	**0.02 **(0.16)	**0.01 **(0.18)	**0.04 **(0.15)
mapping2.eye	24/234532/0.78	0.27 (0.12)	0.07 (0.16)	0.43 (0.09)	0.70 (0.06)	0.19 (0.14)	0.34 (0.12)
mapping2.imaginal disc	21/154400/0.69	0.16 (0.10)	0.20 (0.09)	**0.00 **(0.29)	0.09 (0.12)	0.57 (0.08)	0.63 (0.03)
mapping2.mesoderm	6/47232/0.74	0.62 (0.08)	**0.00 **(0.24)	0.26 (0.11)	0.12 (0.13)	**0.00 **(0.22)	0.00 (0.24)
mapping2.neuronal	12/86317/0.79	0.49 (0.09)	0.16 (0.12)	0.90 (0.05)	0.91 (0.05)	0.24 (0.12)	0.72 (0.07)
mapping2.reproductive system	9/111351/0.85	**0.00 **(0.30)	0.37 (0.08)	0.59 (0.05)	**0.02 **(0.15)	0.16 (0.14)	**0.00 **(0.20)
mapping2.wing	12/84154/0.77	**0.02 **(0.17)	0.14 (0.12)	0.89 (0.04)	0.28 (0.10)	0.14 (0.13)	0.18 (0.11)
mapping3.adult	12/54278/0.77	**0.00 **(0.20)	**0.03 **(0.14)	**0.05 **(0.13)	0.34 (0.09)	**0.01 **(0.20)	**0.05 **(0.13)
mapping3.larva	33/340094/0.78	0.97 (0.03)	0.06 (0.11)	1.00 (0.01)	0.24 (0.09)	**0.05 **(0.14)	0.97 (0.03)

^*∗*^The number of sequences, total length of sequences, and maximum sensitivity of sequences on a subdataset.

^†^The empirical *p* value (and sensitivity) of a method's predictions. Statistically significant predictions (*p* value ≤ 0.05) are shown in bold.
